# Scale-up influences and definitions of scale-up ‘success’: evidence from globally scaled interventions

**DOI:** 10.1093/tbm/ibae063

**Published:** 2025-02-11

**Authors:** Harriet Koorts, Jiani Ma, Samuel Cassar, Adrian Bauman, Mark Lawrence, Harry Rutter, Jo Salmon

**Affiliations:** School of Exercise and Nutrition Sciences, Institute for Physical Activity and Nutrition (IPAN), Deakin University, Geelong 3125, Australia; School of Exercise and Nutrition Sciences, Institute for Physical Activity and Nutrition (IPAN), Deakin University, Geelong 3125, Australia; School of Exercise and Nutrition Sciences, Institute for Physical Activity and Nutrition (IPAN), Deakin University, Geelong 3125, Australia; Prevention Research Collaboration, Sydney School of Public Health, The University of Sydney, Sydney, NSW 2006, Australia; The Australian Prevention Partnership Centre, Sax Institute, Sydney, NSW 2037, Australia; School of Exercise and Nutrition Sciences, Institute for Physical Activity and Nutrition (IPAN), Deakin University, Geelong 3125, Australia; Department of Social and Policy Sciences, University of Bath, Claverton Down, Bath, BA2 7AY, UK; School of Exercise and Nutrition Sciences, Institute for Physical Activity and Nutrition (IPAN), Deakin University, Geelong 3125, Australia

**Keywords:** scaling up, implementation, population, adoption, dissemination

## Abstract

The World Health Organization ExpandNet framework for scaling up contains key recommendations to support the scaling of health interventions globally. Despite being widely used, it is not known how the framework informs intervention scale-up nor how ‘successful’ scale-up is defined. Using data from the Scaling Up InTErventions’ study, this paper assessed adoption of framework components using an international sample of scaled-up physical activity and nutrition interventions, and explored individuals’ definitions of scale-up ‘success’. An online survey with academic, community, and government representatives involved in scaling physical activity and nutrition interventions globally. Survey questions (*n* = 27) corresponded to 32 components of the ExpandNet framework, reflecting four core areas: (i) intervention; (ii) user organization; (iii) resource team; and (iv) scale-up strategy. Data were analysed descriptively and qualitative free-text survey responses coded thematically. In total, 62 survey responses were obtained [academia (*n* = 32), community (*n* = 20), and government (*n* = 10)], corresponding to 35 scaled-up interventions. Only 8% of participants reported all 32 framework components during scale-up. Four core elements (containing eight themes) underpinned successful scale-up: (i) **scaling inputs** (e.g., sustained partner buy-in); (ii) **scaling outputs** (e.g., sustained, quality implementation); (iii) **scaling outcomes** (e.g., increased and equitable reach, improved organization and system capacity) and; (iv) **scaling context** (e.g., partner mental models, and a context-specific construct). There is no universal definition of successful scale-up. We propose core elements of ‘successful scale-up’ that could be used as criteria for scale-up planning and evaluation, and are applicable to other areas of public health.

## Teaser

Influences on scale-up and definitions of scale-up ‘success’ that include scaling inputs, outputs, outcomes, and context.

**Implications**

**Practice:** Discussions surrounding scale-up ‘success’ should occur early, and throughout, implementation and scale-up processes, including all of those involved in or expected to be impacted by scale-up processes and outcomes.

**Policy:** Criteria and outcomes within our proposed key elements of successful scale-up could usefully inform planning actions and evaluation measures, including assessing scale-up.

**Research:** Researchers need to capture how scale-up success is understood and defined by those involved in delivering or supporting population interventions, and use this to guide planning and evaluation of real-world research.

## Introduction

Physical inactivity, poor nutrition, and obesity have reached pandemic proportions worldwide [1–3]. Effective and sustainable policies and interventions implemented at scale are necessary to achieve equitable population shifts in health behaviours. The World Health Organization (WHO) defines scale-up as ‘the deliberate effort to increase the impact of successfully tested health interventions so as to benefit more people and to foster policy and program development on a lasting basis’ [4]. Historically, in physical activity and nutrition research, there has been a substantial disconnection between the development and testing of efficacious interventions on a small scale, and the system-level embeddedness that is required for sustainable health impact at the population level [5]. In 2018, the WHO launched the Global Action Plan on Physical Activity [6], which places explicit emphasis of the need for country-level system reforms to enable scalable solutions to physical inactivity worldwide. The United Nations Decade of Action on Nutrition (2016–25) focuses on national, multisectoral approaches. Sustainable and effective at-scale implementation of interventions is a core objective for many policymakers, practitioners, and researchers. However, the reality is that few physical activity and nutrition interventions [5, 7], and public health interventions more broadly, reach this level of implementation or institutionalization.

The scale-up of population health interventions involves a diverse set of pathways that may not adhere to proposed processes [8], and decisions to scale interventions can also occur opportunistically among policymakers [9]. The complex and dynamic influences on scale-up processes [10] mean that possessing an ideal context or ‘prerequisites’ for scale-up (e.g. evidence-based, addressing a community need) will not necessarily lead to successful scale-up outcomes [11]. Reach is often over-emphasized as an indicator of successful scale-up and marker of impact [12], and in public health is often overestimated [13]. Thus, standard processes of scale-up assessment risk underestimating the range of resources—‘soft infrastructure’—that are necessary for population health improvement [14]. Despite increasing urgency to improve the effectiveness and sustainability of scale-up efforts [6], there is little generalizable knowledge of what is required to successfully scale interventions that target physical activity and nutrition.

To support the scaling up of health interventions globally, the WHO, in collaboration with the ExpandNet group, developed a framework for action (e.g. [4, 15]). Specifically, in 2009, the WHO ExpandNet framework for scaling up [16] was introduced, and comprises four key principles for enhancing scale-up which relate to the intervention (i.e. components are perceived as new in a particular context), testing of the intervention (i.e. evidence-based), that scaling up involves deliberate efforts (i.e. a guided process as opposed to spontaneous diffusion), and that it fosters program and policy development on a lasting basis (i.e. developing and establishing political support). It also incorporates six core areas for consideration during scale-up: (i) intervention attributes (e.g. features that increase the likelihood of an intervention being successfully transferred, such as credibility, relevance, and compatibility); (ii) user organization attributes (e.g. organizational characteristics such as perceived need and implementation capacity); (iii) environment attributes (e.g. opportunities in the environment to minimize constraints or accelerate institutionalization, such as the policy context and bureaucracy); (iv) resource team attributes (e.g. features that increase likelihood of attaining scale-up goals, such as a unifying vision and effective leadership); (v) scale-up strategy (e.g. plans and actions necessary to establish intervention such as advocacy strategies, and monitoring and evaluation); and (vi) planning and management (e.g. strategic monitoring and consistent attention to the scaling up process, and maintaining an appropriate balance among elements of the scaling up system, such as recognizing when trade-offs are necessary) [16]. The framework includes five strategic areas for consideration prior to and during scale-up including dissemination and advocacy, organizational process, costs/resource mobilization, and monitoring and evaluation.

In 2018, the PRACTical planning for Implementation and Scale up (PRACTIS) Guide [7] was developed, to integrate evidence from implementation science with recommendations for scaling up, to produce an evidence-based practical tool for researchers, practitioners, and policymakers to prospectively plan or retrospectively evaluate implementation and scale-up. As with other tools in the field (e.g. [17, 18]), the WHO ExpandNet framework [16] and the PRACTIS Guide [7] provide a series of recommendations and questions regarding the broader system context when scaling health interventions. However, it is not known whether recommendations from these frameworks and tools are adopted in practice, which factors influence the real-world scale-up of physical activity and nutrition interventions worldwide, or how ‘successful scale-up’ is defined and operationalized in practice. Whilst there is no formal definition of successful scale-up, in physical activity promotion it has been referred to as system-level embeddedness of interventions for ongoing health impact [5]. Nonetheless, interpretations of ‘successful scale-up’ are varied, and how to capture different conceptualizations of ‘success’ through evaluation is unclear [19]. The current lack of guidance has led to tensions among researchers, practitioners, and policymakers surrounding the ways evidence is generated for knowledge translation, individuals’ beliefs about how scale-up should occur, and thereby differing interpretations of scale-up ‘success’ [19].

In 2018, the ‘Scaling Up InTErventions’ (‘SUITE’) project was conducted to understand factors influencing the scale-up of a sample of physical activity and nutrition interventions globally, from the perspectives of researchers, practitioners, and policymakers who may play very different roles in the scale-up process [11]. A central aim of the SUITE project was to explore the WHO ExpandNet framework components among previously scaled physical activity and nutrition interventions worldwide, and identify specific factors influencing scale-up globally that could extend the PRACTIS Guide [7] recommendations and explore what is conceptualized as ‘successful scale-up’. In this paper, our aims were to: (i) identify key characteristics (e.g. funding source and roles) of an international sample of scaled-up physical activity and nutrition interventions; (ii) assess presence of the WHO ExpandNet framework components among these scaled-up interventions, and how adoption varied at different phases of scale-up; (iii) identify core criteria underpinning definitions of successful scale-up; and (iv) explore differences in the perspectives of researchers, practitioners, and policymakers involved in scaling up.

## Materials and Methods

### Study design

An online survey was conducted with individuals involved in scaling up interventions, representing academic, government, and NGO, using data from the ‘SUITE’ study [11]. The WHO ExpandNet framework for scaling up [16] provided the theoretical framework underpinning the online survey design. A detailed description of the study methods has been published previously [11]. In the SUITE study, we defined interventions as ‘a set of actions with a coherent objective to bring about change or produce identifiable outcomes’ (30). We included only researcher and non-researcher (e.g. community/government) led programs and initiatives, and excluded those described as a policy, strategy or government regulation. Due to the lack of a formal definition, we classified ‘successfully scaled-up’ interventions as those that had achieved state or national roll out, without them necessarily having demonstrated any effectiveness on a health or behavioural outcome.

### Identifying scaled-up interventions

#### Eligibility criteria

Interventions were required to have: (i) a primary objective of improving physical activity and/or nutrition in accordance with global guidelines [20, 21], and (ii) publicly available information on the intervention, scale-up process, and program lead contact details, to determine relevance to the study. Interventions were required to have been implemented at a state, territory/region, or national level during 2010–18 (studies in Australia) and 2010–19 (studies outside of Australia), or planned for implementation at a state, territory/region, or national level during 2017–18 (studies in Australia) or 2017–19 (studies outside of Australia). Interventions in Australia were also required to have been rolled out, or planned for roll out, with a state or Federal government, as opposed to only via an industry or NGO.

The inclusion criteria differed slightly for Australian versus non-Australian interventions. This was due to the availability of additional project funding in 2019, which meant that we could expand the study from solely investigating scale-up of Australian interventions, to include global interventions. The date range therefore differed as searches for Australian interventions occurred in 2018 (during the initial project funding period), whereas the searches for non-Australian interventions took place in 2019 (after additional project funding). In addition, to accommodate any differences in the funding approaches used to scale interventions within and outside of Australia, for interventions scaled-up outside of Australia we did not exclude those scaled up only by an NGO or industry (thus excluding government involvement). Intervention funding information was collected via the survey for the purposes of reporting and comparison. The opportunity to expand the scope of the study did not alter the underpinning objectives and data analysis methods.

#### Search strategy

Interventions were identified via peer-reviewed literature search, using pre-specified search terms and online databases (e.g. EBSCO host), an advanced Google search, and subject matter expert recommendation, which are described in detail elsewhere [11]. Database searches were conducted from 01 January 2010 to 12 December 2018. For consistency and to ensure coverage of the appropriate literature, search strings were adapted from those used by Reis *et al*. [5], and a research librarian was consulted during their development and testing. Preliminary screening was conducted immediately after online and grey-literature searches to remove duplicates, non-relevant programs/website links, and those not planned or currently scaled up. Remaining interventions were then screened against the study inclusion criteria below. A grey-literature search was conducted via Google to capture any government or non-government led initiatives that were not reported in the online peer-reviewed databases. In instances where the program link or publicly available documentation provided insufficient information relating to the study, further Google searches were conducted (e.g. via the program’s website) to determine eligibility. Online databases and grey-literature search strings are shown in Additional File 1.

#### Final screening

Ninety-eight interventions were assessed against the study inclusion criteria by four members of the research team (screened interventions listed in Additional File 2). Sixty-three interventions were excluded, mostly due to being unable to recruit participants or a lack of publicly available information. This step resulted in 35 interventions that met the study inclusion criteria, which were included in the final analyses.

### Participants and recruitment

Participants included key individuals working in academia (e.g. University-based academics responsible for designing/testing the intervention), government (e.g. policymakers/civil servants involved in government adoption and/or implementation of the intervention), and NGOs (e.g. industry or community-based organizations that had a significant role in the scale-up process). Participants were recruited via purposive sampling to identify individuals named in publications/reports associated with each intervention, followed by snowball sampling to identify additional individuals who had a significant role in scaling up, but may have been missed during purposive sampling.

### Procedure

All participants were invited via email to participate in an online survey lasting up to 30 minutes. All participants were sent a plain language description of the project, and provided online consent prior to commencing the survey.

### Measures

The online survey contained 27 core questions, including 12 follow-on sub-questions, derived from the WHO report ‘20 Questions for Developing a Scaling up Case Study’ [22]. All participants were asked to respond to the core survey questions. Participants were only asked additional follow-on sub-questions based on their previous response (yes/no) to the core question. For the purposes of this study, we included the four key principles and four of the six core areas of the WHO ExpandNet framework in the survey (excluding ‘environment attributes’ and ‘planning and management’) (survey included in Additional File 3). The WHO core area ‘environment attributes’ was instead captured in interviews as part of the larger SUITE study [11], whereas the ‘planning and management’ domain was excluded as it was not relevant to the study aims. These key principles and core areas corresponded to all 32 discrete framework components, which informed the data collection and analysis. Table 1 presents example survey questions and their application to the four core areas of the framework. Survey items included yes/no responses, open-ended questions, or were rated on a five-point Likert scale from strongly disagree (i) to strongly agree (v), including a ‘don’t know’ option. Core survey questions related to descriptive characteristics (eight questions), intervention attributes (seven questions), user organization attributes (one question), resource team attributes (one question), scale-up strategy (five questions), and general reflections (e.g. major facilitators experienced during scale-up) (five questions) (Table 1).

**Table 1:** Application of the four selected core areas of the WHO ExpandNet framework for scaling up to survey questions

WHO core area	Description^a^	Example survey question
Intervention attributes (7 core questions; 2 subquestions)	The intervention designates what is being scaled up. It may refer to interventions tested in pilot or experimental projects, or new practices that are not focussed on services. The concept of ‘innovation’ means that the practices are new (actual or perceived) in the local contexts where they are being introduced. It can include a package of interventions (not only a single intervention) and new service components and managerial processes necessary for implementation.	How much do you agree with the following statement: The initiative was tested in a research trial and was shown to be feasible for delivery in the target setting?
User organization attributes (1 core question)	The institutions or organizations that seek or are expected to adopt and implement the intervention on a large scale. Can include a public sector health service system, a NGO or alliance, a network of private, commercial sector providers, or a combination of such institutions	How much do you agree with the following statement: The user organization(s) had appropriate implementation capacity to deliver the initiative successfully?
Resource team attributes (1 core question; 2 subquestions)	Individuals and organizations that seek to promote and facilitate wider user of the intervention. The resource team serves as a catalyst for change and provides guidance and technical assistance to the deliberate efforts to utilize the intervention on a large scale. Can include researchers, program managers, trainers, service providers, community representatives, reproductive health advocates, and policymakers.	How much do you agree with the following statement: The resource team included individuals or organizations that had the capacity to train members of the user organization to support/deliver the initiative appropriately
Scale-up strategy (5 core questions; 6 subquestions)	Plans and actions necessary to establish the intervention in policies, programs, and service delivery. Includes efforts used by the resource team and approaches by the user organization as it responds to these efforts.	During scale-up, were coalitions and networks generated to advocate for changes in policy or laws that were required for successful scale-up?

^a^Core area definitions sourced from Simmons and Shiffman [23].

### Analysis

Quantitative survey data were analysed descriptively using Stata SE 17 (StataCorp LP, College Station, Texas). Differences in the proportion of participants agreeing between groups were tested using Fisher’s exact test, and differences in proportions for dissemination channels and strategies were tested using a McNemar test.

Qualitative free-text survey responses were analysed using a reflexive thematic analysis approach [24]. Following the guidance on reflexive thematic analysis [25], two members of the research team coded the text responses in a collaborative and reflexive manner to develop a nuanced reading of the data (codes). By comparing and contrasting codes, the same two members of the research team generated themes that represent the shared meaning and underlying patterns of codes (i.e. their commonality of level and target of relevance), which were then reviewed and refined to reflect the multiple facets of scale-up success as reported by participants. All participant and organizational level data were anonymized, and results aggregated and presented per the three participant levels were described previously.

## Results

A total of 62 survey responses (49% female) were obtained (representing academic [*n* = 32], community [*n* = 20], and government [*n* = 10] participants) (Additional File 4). Survey responses corresponded to 35 scaled-up interventions (see Additional File 2 for descriptive information). Interventions targeted improvements in nutrition (*n* = 8), physical activity (*n* = 9), and a combination of both (*n* = 18). Interventions scaled up solely in one country included: Australia (*n* = 16), USA (*n* = 9), The Netherlands (*n* = 4), Belgium (*n* = 1), UK (*n* = 1), Spain (*n* = 1), and Canada (*n* = 1). One intervention was scaled across Fiji, Tonga, New Zealand, and Australia.

### Characteristics of scaled interventions

#### Scale-up funding source

Governments were a primary source of funding for most interventions (*n* = 32, 91%). Six interventions had multiple sources of primary funding, which included Government and NGOs (*n* = 2), Government and charity/philanthropic organizations (*n* = 1), industry and NGOs (*n* = 1), Government and industry (*n* = 1), and a combination of Government, industry, NGOs, and charity/philanthropic organizations (*n* = 1). One intervention was funded solely by an international agency, and one was funded solely by a charity/philanthropic organization. For those interventions supported by Government, the perceived main reasons for support included: (i) addressed a priority area of a specific Government department (*n* = 29, 71%); (ii) aligned with a strategic priority of the Government (*n* = 25, 61%); (iii) addressed a key need in the community (*n* = 22, 54%); (iv) contributed towards meeting a relevant target (*n* = 18, 44%); (v) an opportune policy window opened, or the political climate was ‘right’ (*n* = 14, 34%); (vi) perceived to be technically and financially feasible within existing capacity and resource constraints (*n* = 16, 39%); (vii) as a result of internal/external advocacy (*n* = 13, 32%); and (viii) aligned with existing institutional structures and historical policy trajectories (*n* = 9, 22%).

#### Scale-up role

Participants’ primary roles with the intervention included: ‘overseeing or coordinating/supporting initiative delivery in practice’ (*n* = 38, 61%), ‘design/development of intervention components/strategies’ (*n* = 37, 60%), ‘evaluating impact or delivery of the initiative in practice (e.g. non-research trial)’ (*n* = 37, 60%), ‘testing in a research trial/pilot study’ (*n* = 34, 55%), ‘advocacy or support for initiative implementation in practice’ (*n* = 30, 48%), ‘decision-making process to adopt or fund the initiative at a state/territory/national level’ (*n* = 17, 27%), ‘provision of funds to support implementation or scale-up in practice’ (*n* = 13, 21%), and ‘provision of funds to support research relating to the initiative’ (*n* = 8, 13%).

### Presence of the WHO ExpandNet framework components during scale-up

**Figure 1:**
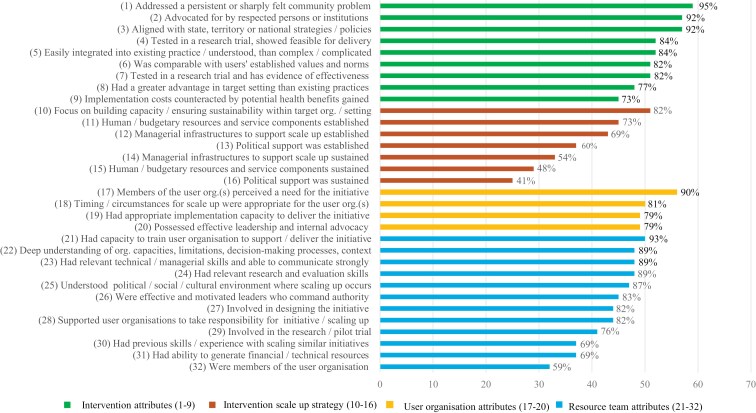
Presence of the WHO ExpandNet framework components during scale-up

Figure 1 presents participant-reported presence of the WHO ExpandNet framework components during scale-up. Whilst the 32 framework components (Figure 1) are recommended by the WHO, only 8% (*n* = 4) of participants reported that all components were present during scale-up. Presence of the different WHO ExpandNet framework components varied by framework core area (intervention attributes, intervention scale-up strategy, user organization attributes, and resource team attributes). For example, only 41% of participants reported that political support was sustained (core area: intervention scale-up strategy), whereas 95% of participants reported that the intervention addressed a persistent or sharply felt community problem (core area: intervention attribute) (Fig. 1).

Additional File 5 presents participant agreement to the presence of the 32 different components of the framework during scale-up. Across the majority of the components, there was general consistency between participant groups. Significant differences between groups were only observed for three components relating to the intervention scale-up process (Additional File 5); (i) managerial infrastructures to support scale-up established, (ii) managerial infrastructures to support scale-up sustained, and (iii) human/budgetary resources and service components sustained. Specifically, all government participants reported that managerial infrastructures to support scale-up were established prior to scale-up (e.g. during scale-up planning phases or when government support/funding was being sought), whereas community and government participants were significantly more likely, compared to academics, to report that managerial infrastructures and human/budgetary resources were sustained after scale-up (Additional File 5). For intervention attributes, 36% (*n* = 22) of participants reported that all nine components were present during scale-up. For the intervention scale-up strategy, 42% (*n* = 26) of participants reported that all four components were present *prior to* scale-up, whereas only 31% (*n* = 19) of participants reported that all three components were present following scale-up. Of note, 38% (*n* = 23) of participants reported that none of the components were present after scaling up. In total, 63% (*n* = 39) of participants reported that all four components relating to user organization attributes were present prior to scale-up. The majority of participants (89%, *n* = 55) reported that a resource team was involved in facilitating scale-up (81% academics, 95% community, and 100 % government participants); however, only 28% (*n* = 15) of participants reported that all 12 items related to the resource team attributes were present.

#### Scale-up approach

Approaches to scale-up varied and often changed during scaling up (i.e. approaches prior to and during scale-up). Of respondents, 37% (*n* = 22) reported that coalitions and networks were generated to advocate for changes in policy or laws to enable successful scale-up. Of these 22 respondents, 96% (*n* = 21) reported that this involved individuals or organizations outside of the government health sector. The scale-up approach included factors related to: (i) dissemination channels prior to and during scale-up; (ii) dissemination strategies prior to and during scale-up; and (iii) strategies used to enhance scale-up within user organizations. Table 2 presents differences in the reported use of dissemination channels and strategies prior to and during scale-up, and strategies used to enhance scale-up within user organizations.

**Table 2:** Differences in adoption of WHO ExpandNet recommended dissemination channels and strategies, prior to and during scale-up

	Prior to scale-up; *N* (%)	During scale-up; *N* (%)	*P*
Dissemination channels	Max *n* = 58	Max *n* = 58	
Policy makers	42 (72)	38 (66)	0.28
NGOs or community-based organizations	30 (52)	42 (72)	0.004
Organizations or settings who would deliver the intervention	42 (72)	51 (88)	0.04
Community members/advocacy groups	20 (35)	36 (62)	0.001
Target recipients (i.e. end users) of the initiative	28 (48)	44 (76)	0.002
Other	5 (9)	4 (7)	0.65
Dissemination strategies	Max *n* = 55	Max *n* = 55	
Messages were tailored and used a format for each audience	38 (69)	48 (81)	0.16
Data were presented clearly, concisely, and in a timely manner, so that it was relevant and useable	43 (78)	50 (85)	0.41
Repeated messages integrated into established communication networks of user organizations	30 (56)	45 (76)	0.03
Barriers to effective communication were recognized, communication/marketing specialists used	26 (47)	37 (63)	0.11
Other	5 (9)	2 (3)	0.16
Strategies to enhance scale-up in user organizations	Max *n* = 59		
Providing resources to support implementation (e.g. information packs, toolkits)	54 (92)		
Providing face-to-face training of target organizations and employees	51 (86)		
Newsletters with information on intervention and/or implementation experiences	43 (73)		
Monitoring and providing feedback to organizations on implementation performance	42 (71)		
Using online networks to communicate or support local implementation efforts	38 (64)		
Remote or online technical assistance/support services	34 (58)		
Providing online training of target organizations and employees	32 (54)		
Other	4 (7)		

McNemar test used to assess differences in proportions for dissemination channels and strategies, prior to and during scale-up.

(i) Dissemination channels prior to and during scale-up

Of the six possible dissemination channels, most respondents (*n* = 40, 70%) reported using between 2 and 4 channels prior to scale-up. During scale-up, most respondents (*n* = 46, 79%) reported using between 3 and 5 channels. There were significant differences in the types of dissemination channels used at the different phases of scale-up (Table 2). During scale-up, compared to prior to scale-up, an estimated 21% (95% CI: 6%–36%) more respondents reported using NGOs or community-based organizations, 16% (95% CI: 0%–31%) more respondents used organizations or settings that would ultimately deliver the intervention, 28% (95% CI: 11%–44%) more respondents used community members/advocacy groups, and 28% (95% CI: 9%–46%) more respondents targeted recipients of the intervention.

(ii) Dissemination strategies prior to and during scale-up

Of the five possible dissemination strategies, most respondents (*n* = 41, 75%) reported using between 2 and 4 strategies prior to scale-up. During scale-up, most respondents (*n* = 41, 70%) reported using three or four strategies. There was a significant difference in the use of repeated messages into established communication networks of the user organization (Table 2). During scale-up, compared to prior to scale-up, an estimated 19% (95% CI: 1%–36%) more respondents reported integrating repeated messages into established communication networks of the user organization.

(iii) Strategies to enhance scale-up in user organizations

Of the eight possible strategies to enhance scale-up in user organizations, most participants (*n* = 51, 86%) reported using between 4 and 7 strategies. The most commonly reported strategy (92% of participants) was the provision of resources to support implementation (Table 2). Whilst online delivery could enhance the reach of interventions at scale; 86% of participants reported face-to-face training as a strategy to enhance scale-up, and the least reported strategy included online training delivery (54% of participants).

#### Scale-up evaluation

Most participants stated that the intervention had been evaluated at scale (*n* = 50, 83%), and this included both process evaluation (i.e. intervention reach, adoption) and outcome measures (i.e. target population behaviour change). For those participants that reported why there was no evaluation (*n* = 6), reasons included a lack of funding (*n* = 2), the stage of implementation was too early for evaluation (*n* = 2), or an evaluation was outside of the remit/responsibility of the organization scaling the intervention (*n* = 2). In some cases, evaluation findings were used to modify the scale-up process, which included quality improvement, refinement, and adaptation where possible:

…continual improvements have been made to the program over time. Specifically, creating online training modules and applying for continuing education credit for [name of sector] professionals. (Government participant, physical activity and nutrition intervention scaled state-wide)

Reach, adoption and implementation results are used to enhance certain communication or implementation strategies where (financially) possible. However, financial limitations restrain adaptations or reinforcements. (Community participant, physical activity intervention scaled state-wide)

There were mixed responses, but around half (54%, *n* = 14) stated that the evaluation findings had influenced changes to policy or practice. This included interventions becoming embedded as part of routine health services, ongoing support leading to program evolution into digital formats, and scale-up processes being used to inform program roll-outs internationally. Specifically, some participants referred to the use of evidence to influence priority areas of government, including the allocation of funds:

The evaluation findings along with Government monitoring assisted in setting food literacy as a priority area and getting money set aside for program funding. (Community participant, nutrition intervention scaled state-wide)

Outcomes influenced an individuals’ advocacy opportunities and ability to influence policy change:

Results have positioned us to influence both policy and practice. Specifically, we are invited to sit at ‘tables of influence’. I know from previous work that policy change is a far term goal - often takes 10 years or so. (Academic participant, physical activity intervention scaled state-wide)

### Participant definitions of ‘successful scale-up’

**Figure 2:**
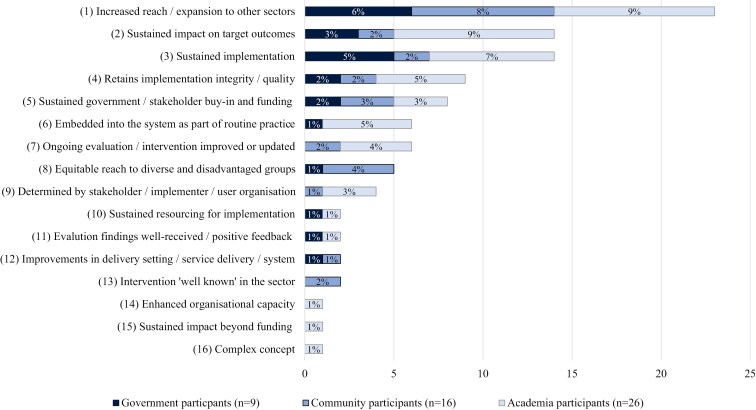
Participant codes (% responses by group) for definitions of successful scale-up (*n* = 16)

Sixteen codes were identified that reflected participants’ definition of successful scale-up (Fig. 2). It should be noted that participants’ responses often include multiple codes.

Whilst some themes were present across all participant groups (Fig. 2), there were differences in the proportion of participants within each group that identified the different themes. For example, almost twice the proportion of government participants, compared to academic participants, referred to the need for high reach and expansion to other sectors (Code 1), sustained implementation (Code 3), and sustained government/partner buy-in and funding (Code 5):

When the program has reached the desired outcomes in relation to population reach (i.e., accessed/implemented by more participants than the original rollout/pilot) and is accepted by that population. (Government participant, physical activity and nutrition intervention scaled state-wide)

When all stakeholders continue to see the benefit to the end-user, and have a commitment to maintain and continuously improve the initiative. When government departments recognise a cost-benefit analysis that is in their favour and provide support for the initiative even if it partially sits in private sector… (Community participant, physical activity intervention scaled up nationally)

Only government and community participants referred to the importance of the intervention reaching diverse and disadvantaged groups to address inequities (Code 8):

The intervention is accessible to all interested parties regardless of socioeconomic status, cultural background or geographic location, but especially to those that are at the most risk of the problem the intervention is aiming to address. (Community participant, nutrition intervention scaled nationally)

[Intervention] delivered universally with tailoring to address inequities. High reach and impact that does not vary by relative social disadvantage (Government participant, physical activity and nutrition intervention scaled state-wide)

Almost three times the proportion of government and academic participants, compared to community participants, referred to the intervention needing to be implemented at scale whilst sustaining impact on target outcomes (Code 2). Yet only government and academic participants described the need for interventions to become embedded into existing systems, so they become part of routine practice (Code 6):

I think it is successfully scaled up when it has been integrated in daily practice - it has become normal… (Academic participant, physical activity and nutrition intervention scaled nationally)

Only community participants referred to the intervention needing to be well-known in the sector (Code 13):

…When the name of the initiative is second nature and just rolls off the tongue of all key players. (Community participant, physical activity intervention scaled up nationally)

Both academic and government participants referred to the importance of research and evaluation findings being well-received through positive feedback (Code 11):

…success is also defined by feedback and relationships, and the program has and continues to have very positive feedback from educators and stakeholders. (Government participant, physical activity and nutrition intervention scaled state-wide)

Only academic participants described ‘success’ as a dynamic and complex concept (Code 16) that is determined by the individual and outcome of interest (Code 9):

Successful scale up is a complex concept - on the one hand scale up may need to ensure program integrity is maintained, but scale up is across different units, social settings and geographic locations, and a program would need to be adapted to these environmental circumstances in order to be relevant, but through that adaptation its integrity is compromised. Also, different units adopting the one program may do so for different purposes, and hence the outcomes of success would be defined differently. (Academic participant, nutrition intervention scaled nationally)

Success is the term most difficult to define as I believe success is defined in collaboration with stakeholders for each initiative. The academic response might be that success is achieved when an initiative is replicated in multiple non-research settings with optimal levels of implementation and high-level perceived feasibility from stakeholders while enhancing organisations’ capacity to sustain future implementation. (Academic participant, physical activity intervention scaled up state-wide)

### Key elements of successful scale-up

**Figure 3:**
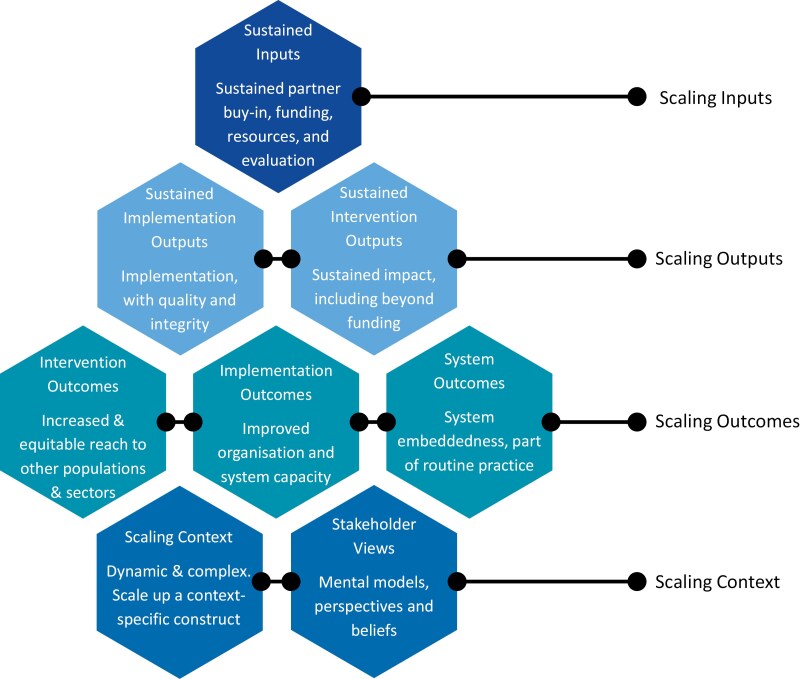
Key elements underpinning ‘successful scale-up’

Eight themes were identified based on the 16 codes (Fig. 2) that represented participants’ definitions of scale-up success: Theme 1: Sustained partner buy-in, funding, resources, and evaluation (Codes, 5, 7, and 10); Theme 2: Sustained implementation, with quality and integrity (Codes 3 and 4); Theme 3: Sustained impact, including beyond funding (Codes 2 and 15); Theme 4: Increased and equitable reach to other populations and sectors (Codes 1 and 8); Theme 5: Improved organization and system capacity following implementation (Codes 12 and 14); Theme 6: System embeddedness and part of routine practice (Code 6); Theme 7: Partner mental models, perspectives, and beliefs (Codes 9, 11, and 13); and Theme 8: Dynamic, complex, and context-specific construct (Code 16). Figure 3 presents the key elements (eight themes) underpinning definitions of successful scale-up, derived from the open-ended survey question, including how these correspond to scaling inputs, outputs, outcomes, and context.

Scaling inputs includes the sustainability of external resources and partner relationships that support or enable scaling up. Specifically, sustained partner buy-in and sustained funding, sustained provision and availability of resources, and ongoing monitoring and evaluation for refinement and decision-making purposes. Resources can include financial support from funders, and individual/organization buy-in and advocacy. Scaling outputs refers to activities that support the desired scaling outcomes. This includes sustained intervention use for ongoing impact beyond funding periods, and implementation that retains quality and integrity to maximize impact.

Scaling outcomes includes intervention, implementation, and system-level outcomes. For interventions, this includes positive and equitable impacts from actions targeting health behaviours (on target and wider population), and positive impacts of the scaling process itself (i.e. beneficial expansion for increased reach). It also includes impacts beyond the target population and any positive outcomes that arise from implementation, such as increased capacity or capabilities of organizations to deliver change as a result of implementation. System outcomes include embeddedness of interventions and implementation, and the institutionalization of actions at organizational and/or systems levels.

Scaling context refers to the holistic interpretation and conceptualizations of scale-up that are to be expected across the diversity of individuals, settings, and sectors involved. This includes the mental models and subjective judgement of those involved in scaling up. For example, the perspectives (e.g. receipt of positive feedback regarding the intervention), perceptions (e.g. the intervention is perceived to be well-known), and beliefs (e.g. the intervention has impact) among individuals and organizations involved. It also includes recognition of the complex, interrelated, and dynamic interactions that occur during the scaling process, and that definitions of scale-up are dependent on partners, setting, and context, which can change over time.

## Discussion

The purpose of this study was to explore the factors, and timing of factors, leading to successful scale-up of physical activity and nutrition interventions globally, participant interpretations of ‘successful scale-up’, and the extent that components of the WHO ExpandNet framework had been adopted in included interventions. We addressed these aims by exploring differences in the perspectives of researchers, practitioners, and policymakers involved in scaling up.

Our findings showed that all four core areas of the WHO ExpandNet framework were relevant to the scaling up of interventions; however, only a minority of participants (8%) reported that all 32 framework components were present. The authors of the ExpandNet framework do not suggest that all components are mandatory for effective scale-up; they describe these factors as key for consideration during planning where possible. However, our findings highlight that scale-up can be achieved based on a vast array of contributing factors. This is consistent with previous research that has shown that scale-up processes are highly diverse and need not follow prescribed or sequential stages [8].

Our findings also showed that approaches to scale-up varied and changed over the course of scaling up. For example, a greater number and diversity of channels for dissemination were used during rather than prior to scale-up. This suggests that dissemination channels may become increasingly important once scale-up had commenced. Whereas, of the five reported dissemination channels, only the use of policymakers as a dissemination channel was utilized over the course of scale-up. This is consistent with previous research that has identified policymakers as having a vital role in decisions to roll out an intervention [9, 26], and the influence of changes in the political environment on scale-up outcomes [27]. Around a third of participants used coalitions and networks for advocacy, and the majority of these involved government sectors outside of health. This finding adheres to WHO recommendations [16] and is unsurprising given that scale-up in physical activity and nutrition typically involves sectors outside of health [5, 28]. In terms of strategies used to enhance scale-up within user organizations, the second most commonly reported strategy included face-to-face training. The least reported strategy included online training delivery to organizations or employees. Online delivery may increase the reach of physical activity and nutrition interventions at scale in terms of the number and diversity of participants [29]; however, the impact of online training/delivery on subsequent effectiveness and sustainability of interventions at scale is likely to be context specific. In this study, we were not able to ascertain in what ways different strategies were perceived to enhance scale-up in user organizations. Ascertaining the impact of different strategies on scale-up outcomes (i.e. intervention reach versus sustainable intervention impact) would aid the selection and use of strategies for key outcomes.

Around half of the participants reported that scale-up was evaluated, and that evaluation findings influenced practice and policy (e.g. interventions becoming embedded in routine health services or influencing government priority areas). Yet, we also found that ‘successful scale-up’ was both a subjective (based on perceptions) and potentially objective (quantifiable) construct among participants; although quantifiable interpretations were inferred (i.e. participants referred to ‘increased reach’, which typically includes a rate of uptake). This may reflect the fact that we currently lack recommendations for how to measure and evaluate scale-up success [19]. How success is defined often underpins what is collected, measured, and used for decision-making purposes from evaluations [19]. It is not known from our results whether the evaluations of the interventions included in this study aligned with the criteria of scale-up success for those involved. While it is known that different groups value and use different sources of evidence for decision-making [30], and given the diversity of scale-up roles (i.e. intervention design to scale-up funder) we included in this study, it is not surprising that interpretations of scale-up success varied. However, this does have implications for the evaluation of scale-up in population health. Firstly, it reinforces the need for early awareness and agreement of what ‘success’ means to different groups, and secondly, that this interpretation may evolve over time and is not a static concept.

Although there was general consistency between participant groups in their perceptions of what led to successful scale-up (e.g. types of strategies needed), there was no single, consistent definition of successful scale-up and participants’ responses were described as a multifaceted construct. Critically, our findings showed that ‘successful scale-up’ included an array of factors that were beyond solely the intervention and its impact on health (e.g. sustained government resources and buy-in for implementation). Our results showed that successful scale-up need not be determined based on the presence of all themes in Figure 3. Multiple features existed within each theme, and the content of Figure 3 is not intended to be an exhaustive list. Rather, scale-up was considered ‘successful’ by participants based on a combination of, or a single factor within, any one theme (i.e. the intervention was ‘well-known’; scaling context). Based on these findings, including prior research in this area [10, 19, 26], we suggest that ‘successful scale-up’ is a practice-led holistic construct that should be interpreted uniquely based on the scaling context and actors involved.

Scale-up paradigms typically align closely with ecological frameworks and systems perspectives [31], as the external conditions or structures of existing systems within which interventions are implemented and scaled (e.g. contextual factors such as political will, human resources, and partner buy-in) also are mechanisms of scale-up outcomes and population impact [11]. The PRACTIS guide [7] acknowledges that successful scale-up can depend on the interaction between an intervention’s intensity and the resources available within the delivery setting [32, 33]. Our findings extend the PRACTIS guide, by consolidating core elements and criteria that may be important for ascertaining and interpreting scale-up success. In particular, these elements are a useful frame for Step 1 of the PRACTIS guide [7], which focuses on characterizing parameters of the implementation context with partner organizations, and should include early and ongoing consultation about scale-up ‘success’.

Scalability has commonly been conceptualized as the extent to which an efficacious intervention retains its impact when implemented under less-controlled, real-world conditions among a greater proportion of the eligible population [34]. To date, the focus has been on the intervention or program being scaled and the widening of its reach and sustainability of impact. Increased reach and expansion to other sectors were commonly reported as a criterion for scale-up success in this study, and in some cases, as the only criteria for scale-up success. Both government and community participants in our study acknowledged equity as a key component of scaling up, referring to successful scale-up as reaching diverse and disadvantaged groups. This is a positive finding, since there have been calls for a greater focus on equity in the implementation of interventions [35]. Nonetheless, whilst reach metrics alone are not conducive to population impact [12, 19], expansion into other settings and populations is fundamental to scaling interventions. However, at-scale implementation can also be an adaptive and a non-linear process [36]. The dynamic sustainability framework proposes that intervention adaptation is a continuous improvement process for sustainability [37]. Whilst this study identified that implementation fidelity and integrity still were considered key components of successful scale-up, we also found that participants perceived scale-up to be a dynamic process with interrelated influences. This finding is consistent with recent advancements in the field, which have identified scale-up as a complex and dynamic process [10, 38], shifting thinking away from primarily the ‘characteristics of interventions’ (e.g. replicability of intervention components) as the key denominator for scaling outcomes.

The themes of scaling success we identified are based on real-world scaling experiences in practice. Our findings showed that these themes are interrelated and need not be mutually exclusive. The extent that individuals involved in scale-up adopt the criteria within these elements, is going to directly relate to the actions required to undertake and evaluate scale-up. In terms of the practical application of our findings, we propose that criteria and outcomes within our proposed key elements of successful scale-up (Fig. 3) could usefully inform planning actions and evaluation measures. The proposed elements of successful scale-up could also contribute to planning and assessing scale-up and engaging partner organizations using existing resources (e.g. WHO ExpandNet 20 questions for developing a scaling up case study [22], the PRACTIS guide [7], and Intervention Scalability Assessment tool [39]). Whilst our findings showed that not all factors and success elements need to be present for an intervention to be considered ‘successfully scaled’, and we are not able to infer the importance of any one element over another, we argue that elements relating to sustainable and equitable health improvement are an essential component of scale-up success.

### Strengths and limitations

Major strengths of this study are the inclusion of a diverse range of globally scaled interventions and the collection of data from participants representing multiple roles and sectors related to physical activity and nutrition. We took a ‘reverse translation’ perspective, whereby we studied interventions that were already implemented at scale, to explore the process of how they got there. However, our study is not without limitations. Firstly, we only included interventions scaled up during 2010–19 to ensure we captured more recent scale-up efforts and to increase the potential generalizability of our results to current research. However, this means that some interventions would have been scaled ~10 years ago, and thus it is unclear how participant recall of the scale-up process would have impacted on the results. However, due to the length of time often required for scaling up, we purposely included interventions that represented a combination of those scaled more recently and from an earlier date, in an attempt to minimize this issue. In addition, for participant confidentiality reasons, we did not link the participant responses by intervention to their data in the manuscript. Nonetheless, we still found general consistency in our results, irrespective of the scale-up timelines and intervention type. Secondly, almost half of the included studies were of interventions scaled up in Australia. This was due to the number of international studies that were non-responders or did not provide publicly available information. Whilst the use of an evidence-based scale-up framework (WHO ExpandNet) to inform our data collection instruments and analyses strengthened the robustness and potential generalizability of our findings, future studies may wish to compare any differences between scale-up countries and include interventions targeting other areas of public health. Participant definitions of successful scale-up were also based on free text responses via an online survey. Had we been able to interview participants, we may have gathered more in-depth perspectives and been able to prompt for further exploration of key points. We may have also been able to ascertain which types of data (quantitative or qualitative) and by whom were used to determine different elements of successful scaling (e.g. ‘sustained’ and ‘quality implementation’), which would assist evaluations of successful scale-up in future studies. Future research wishing to explore successful scale-up may benefit from a qualitative research design. Lastly, we were unable to infer the importance of any one framework component over another, nor were we powered to analyse any differences in scale-up between physical activity versus nutrition interventions. Future research would benefit from exploring the relative importance of different factors related to successful scale-up.

## Conclusion

The WHO ExpandNet framework is relevant for scaling up in physical activity and nutrition, yet the relative importance of its factors remains unclear. There is no single definition of successful scale-up. Given the transient and context-specific nature of scale-up, formal indicators defining scale-up success may be neither appropriate nor reflective of the extensive settings and contexts within which interventions are implemented. We identified a practice-led holistic definition that varies by intervention, context, and individual involved. We encourage debate within the community regarding ways that successful scale-up is conceptualized in the field and practice, and encourage that discussions surrounding scale-up ‘success’ occur early, and throughout, implementation and scale-up processes. These discussions should include all of those involved in or expected to be impacted by scale-up processes and outcomes, and be used to inform scale-up planning and evaluation.

## Supplementary Material

ibae063_suppl_Supplementary_File_1

ibae063_suppl_Supplementary_File_2

ibae063_suppl_Supplementary_File_3

ibae063_suppl_Supplementary_File_4

ibae063_suppl_Supplementary_File_5
