# Supplementary material for: Scale-up influences and definitions of scale-up ‘success’: evidence from globally scaled interventions
Source: Transl Behav Med. 2025 Feb 11;15(1):ibae063. doi: 10.1093/tbm/ibae063 (PMC11812037; doi:10.1093/tbm/ibae063)
Supplement: ibae063_suppl_Supplementary_File_1 [file ibae063_suppl_supplementary_file_1.docx]

**Additional File 1.** Online databases and search strings

*Online database search*

‘Australia’ string

via EBSCO host, Academic search complete, Ageline, CINAHL complete, Education Source, Environment Complete, ERIC, Community and mass media complete, Global Health, Health policy ref centre, Medline complete, Political science complete, and Sport Discuss. The search conducted on the 2^nd^ of February, 2018 returned 117 hits using the following search string; (("physical activ*" OR diet* OR nutrition*) AND (intervention* OR program* OR initiative*) ) ) AND AB Australia* AND ( (national OR state OR Victoria OR “New South Wales” OR “Northern territory” OR “Australian Capital Territory” OR “Western Australia” OR “South Australia” OR Tasmania OR Queensland ) N5 implement*) ).

‘International’ string

(("physical activ*" OR diet* OR nutrition*) AND (intervention* OR program* OR initiative*) ) ) AND (scaled-up OR scale-up OR scaling-up OR scalabil* OR reach OR expand* OR institutionalization OR institutionalisation OR roll-out OR rolling-out OR disseminat*). The search, conducted on the 12^th^ of December, 2018, included the following databases; EBSCO host (platform), Academic search complete, Ageline, CINAHL complete, Communication and mass media complete, Education Source, Environment Complete, ERIC, Global Health, Health policy ref centre, Medline complete, Political science complete, and Sport Discuss. This resulted in 11,369 (6,538 after duplicates) which were screened at the title level.

*Grey literature search*

‘Australia’ search

An advanced Google search using only the ‘.gov.au’ domain, the search string ("physical activity" OR diet OR nutrition AROUND 2 implement) (intervention OR program OR initiative) (national OR state OR Victoria OR “New South Wales” OR “Northern territory” OR “Australian Capital Territory” OR “Western Australia” OR “South Australia” OR Tasmania OR Queensland) returned 42,100 hits on the 5^th^ of February 2018. Applying a limiter on the second search further reduced the results to 10,700 when the search focused specifically on the on the ‘.gov.au’ domain. This secondary search was performed to ensure hard to acquire governmental documents, interventions and grey-literature were not missed. SC screened the first 50 results (first five pages) of these Google search strings that resulted in an additional five interventions being added to the catalogue of interventions to be screened.

‘International’ search

The advanced Google search was also expanded for the international search to include scale-up terminology to reduce the search output and concomitantly increase the specificity of results. The search strategy was also opened up to remove the .gov.au domain limiter and thus the final search string reads "physical activity" OR diet OR nutrition and intervention OR program OR initiative and scaled-up OR scale-up OR scaling-up OR scalability OR reach OR expand* OR institutionalisation OR roll-out OR rolling-out OR disseminate. SC screened the first 100 results (first 10 pages), increased to allow for the larger array of interventions internationally.
