# Supplementary material for: Scale-up influences and definitions of scale-up ‘success’: evidence from globally scaled interventions
Source: Transl Behav Med. 2025 Feb 11;15(1):ibae063. doi: 10.1093/tbm/ibae063 (PMC11812037; doi:10.1093/tbm/ibae063)
Supplement: ibae063_suppl_Supplementary_File_2 [file ibae063_suppl_supplementary_file_2.docx]

**Additional File 2.** Descriptive characteristics of scaled up interventions

| **#** | **Intervention name** | **Country** | **Scale up level (State/National**  **/International)** | **Intervention type** | **Target outcome** | **Population and setting** | **Scale up funding source** |
| --- | --- | --- | --- | --- | --- | --- | --- |
| **1** | 10,000 Steps Flanders [1] | Belgium | State | Whole-community walking intervention | PA | Adults, individual, social, and environment level approaches | Government |
| **2** | BEAT IT [2] | Australia | National | Group-based physical activity training with lifestyle education and nutrition sessions. | PA & Nutrition | Adults, Community centres | Government |
| **3** | Choose to Move [3] | Canada | State | Choice-based, activity coach-supported program | PA | Community-dwelling, older adults 60+, community settings | Government |
| **4** | Dutch Obesity Intervention in Teenagers (DOiT) [4] | The Netherlands | National | School-based obesity prevention program | PA & Nutrition | 12-14 year old students, schools | Industry, Non-governmental organisations |
| **5** | Food Sensations for Adults [5] | Australia | State (WA) | Community based group education sessions | Nutrition | Low to middle income adults, community organisations | Government |
| **6** | Go4Fun [6] | Australia | State (NSW) | After school obesity treatment program | PA & Nutrition | Children 7-13 above a healthy weight, Community settings (leisure centres, youth clubs) | Government |
| **7** | Healthy Eating, Activity and Lifestyle Program (HEAL) [7] | Australia | National | Community based lifestyle education program | PA & Nutrition | Adults with obesity or risk of lifestyle diseases, community centres | Government |
| **8** | Health Under Construction [8] | The Netherlands | National | Individual lifestyle intervention for workers in the construction industry | PA & Nutrition | Workplace, Adults | Government, Non-governmental organisations |
| **9** | Heart Foundation Heartmoves [9] | Australia | National | Community based group fitness classes | PA | Adults, fitness facilities, community settings, workplaces, aged-care facilities | Government, Charity/philanthropic entities |
| **10** | Jump-in [10] | The Netherlands | National | Multi-component school-based intervention | PA | Children, Schools | Government |
| **11** | Krachtvoer project [11] | The Netherlands | National | Classroom based lessons administered by teachers and school support | Nutrition | Adolescents, Pre-vocational schools | Government |
| **12** | Life! Taking Action on Diabetes [12] | Australia | State (VIC) | Delivered as a group course or a telephone health coaching service | PA & Nutrition | Adults at risk of type 2 diabetes or cardiovascular disease, structured group sessions | Government |
| **13** | Lift for Life [13] | Australia | National | Resistance training program | PA | Adults with or at risk of type two diabetes, licensed health and fitness businesses | Government, Industry |
| **14** | Live Lighter [14] | Australia | State (WA, VIC, ACT & NT) | Educational mass media campaign | PA & Nutrition | Adults, Mass media and social media | Government |
| **15** | Marathon Kids UK [15] | The United Kingdom | National | School based program with goal-based running sessions | PA | Children, Schools | Charity/philanthropic entities |
| **16** | Mississippi Communities for Healthy Living [16] | USA | State | Interactive cooking classes, demonstrations, showcases | Nutrition | Women from social groups and faith-based organisations, community | Government |
| **17** | Munch and Move [17] | Australia | State (NSW) | Training and resources for early childhood educators | PA & Nutrition | Children aged 0-5 years, Early childhood education and care services | Government |
| **18** | OPAL (Obesity Prevention and Lifestyle) [18] | Australia | State (SA) | Community development and social marketing | PA & Nutrition | Children through families, Community-based | Government |
| **19** | Out-of-School Nutrition and Physical Activity (OSNAP) [19] | USA | State | Multicomponent intervention focusing on practices, environments, and policies | PA & Nutrition | Children, schools | Government |
| **20** | Pacific Obesity Prevention in Communities [20] | Fiji, Tonga, New Zealand and Australia | International | whole‐of‐community obesity prevention intervention | PA & Nutrition | 12-19 year old adolescents, whole community | International agency |
| **21** | Partnership for a Fit Kentucky [21] | USA | State | Multicomponent intervention improving access to healthy foods and physical activity | PA & Nutrition | All ages, early care, schools, workplaces | Government, Non-governmental organisations |
| **22** | PEACH (Parenting Eating and Activity for Child Health) [22] | Australia | State (QLD) | Community-based multi-component group educational sessions | PA & Nutrition | Families with overweight/obese children aged 5-11 years, Community settings (hospitals, schools, universities) | Government |
| **23** | Physical Activity 4 Everyone (PA4E1) [23] | Australia | State (NSW) | Whole-school physical activity program | PA | Adolescents, Disadvantaged secondary schools | Government |
| **24** | Physical Activity, Sports and Heath Plan (PAFES) [24] | Spain | State | Physical activity prescription based on motivational interviewing | PA | Adults (especially those with cardiovascular risk factors), healthcare centres | Government |
| **25** | Project Energize [25] | New Zealand | State | School based program tailored to each schools’ needs | Nutrition | Children, schools | Government |
| **26** | QCWA Country Kitchens Program [26] | Australia | State (QLD) | Community, educational/workshops | Nutrition | All ages, community centres | Government |
| **27** | Rural Restaurant Healthy Options Program [27] | USA | State | Point-of-purchase information | Nutrition | All ages, restaurants | Government |
| **28** | State Physical Activity and Nutrition Program (SPAN) [28] | USA | State | Obesity prevention food service guidelines | PA & Nutrition | All ages, community organisations and worksites | Government |
| **29** | Stephanie Alexander Kitchen Garden [29] | Australia | National | School-based food education program | Nutrition | Primary school children, Primary schools | Government, Charity/philanthropic entities |
| **30** | StrongWomen - Healthy Hearts [30] | USA | National | Cardiovascular disease prevention curriculum | PA & Nutrition | Midlife women, community based | Government |
| **31** | Superhero Foods [31] | Australia | State (WA) | Nutrition education and cooking program | Nutrition | Primary school aged children, schools | Government |
| **32** | Texercise Select [32] | USA | State | 10-week facilitator led program with education and exercise components | PA & Nutrition | Older adults, community settings | Government |
| **33** | TOP Star--Teaching Obesity Prevention in Early Childcare Settings [33] | USA | State | Obesity prevention professional development for childcare providers | PA & Nutrition | Childcare providers, childcare resource and referral agencies | Government |
| **34** | YMCA afterschool programs [34] | USA | State | Mandated physical activity standards for after school care programs | PA | Children, Afterschool programs | Government |
| **35** | YMCA Schools' Breakfast Program [35] | Australia | State (QLD) | School based program including food, equipment and guidance | Nutrition | Children, Schools | Non-governmental organisations, Industry, Charity/ philanthropic entities |

Information in table relates only to scale up period for each intervention. PA - Physical Activity, QCWA – Queensland Country Women’s Association, YMCA - Young Men's Christian Association, Australian states: SA - South Australia, QLD – Queensland, NSW – New South Wales, WA – Western Australia, ACT – Australian Capital Territory, NT – Northern Territory

**References**

1. Dubuy, V., et al., *‘10 000 Steps Flanders’: evaluation of the state-wide dissemination of a physical activity intervention in Flanders.* Health Education Research, 2013. **28**(3): p. 546-551.

2. Penny, B., J. Tuccia, and M.A. Brown, *Beat it: Diabetes lifestyle and physical activity program-The effects and affordability of a 12-week community based, physical activity program for people with or at risk of diabetes.* 2010.

3. McKay, H., et al., *Implementation of a co-designed physical activity program for older adults: positive impact when delivered at scale.* BMC Public Health, 2018. **18**(1): p. 1289.

4. Nassau, F.v., et al., *Exploring facilitating factors and barriers to the nationwide dissemination of a Dutch school-based obesity prevention program "DOiT-": a study protocol.* BMC Public Health, 2013. **13**(1201): p. (19 December 2013)-(19 December 2013).

5. Foodbank Western Australia, T. *The Food Sensations® for Adults Program*. 2020 [cited 2020 28/01/2020]; Available from: <https://www.foodbank.org.au/WA/food-sensations-for-adults/?state=wa>.

6. Welsby, D., et al., *Process evaluation of an up-scaled community based child obesity treatment program: NSW Go4Fun(R).* BMC Public Health, 2014. **14**: p. 140.

7. [*https://www.lifestylemedicine.org.au/content/the-healthy-eating-activity-and-lifestyle-program/*](https://www.lifestylemedicine.org.au/content/the-healthy-eating-activity-and-lifestyle-program/). 18.07.24].

8. Tonnon, S.C., et al., *Process Evaluation of the Nationwide Implementation of a Lifestyle Intervention in the Construction Industry.* J Occup Environ Med, 2016. **58**(1): p. e6-14.

9. Department of Health, T. *Heartmoves*. 2012 [cited 2020 28/01/2020]; Available from: <https://www1.health.gov.au/internet/publications/publishing.nsf/Content/healthy-comm-lgag-att_c-toc~healthy-comm-lgag-att_c-heartmoves>.

10. Meij, J.S.B.d., et al., *A mixed methods process evaluation of the implementation of JUMP-in, a multilevel school-based intervention aimed at physical activity promotion.* Health Promotion Practice, 2013. **14**(5): p. 777-790.

11. Bessems, K.M.H.H., et al., *Appreciation and implementation of the Krachtvoer healthy diet promotion programme for 12- to 14- year-old students of prevocational schools.* BMC Public Health, 2011. **11**(1): p. 909.

12. Dunbar, J.A., et al., *Scaling Up Diabetes Prevention in Victoria, Australia: Policy Development, Implementation, and Evaluation.* Diabetes Care, 2014. **37**(4): p. 934-942.

13. Dunstan, D., G. Cormick, and E. Wolf, *The Lift for Life community-based strength training program for people with or at risk of developing type 2 diabetes—A snapshot evaluation.* Journal of Science and Medicine in Sport, 2010. **12**: p. e212.

14. Morley, B., et al., *Population-based evaluation of the 'LiveLighter' healthy weight and lifestyle mass media campaign.* Health Educ Res, 2016. **31**(2): p. 121-35.

15. Chalkley, A.E., et al., *Marathon Kids UK: study design and protocol for a mixed methods evaluation of a school-based running programme.* BMJ Open, 2018. **8**(5): p. e022176.

16. Connell, C.L., et al., *Mississippi Communities for Healthy Living: Implementing a nutrition intervention effectiveness study in a rural health disparate region.* Contemp Clin Trials, 2015. **42**: p. 196-203.

17. Hardy, L.L., et al., *Munch and Move: evaluation of a preschool healthy eating and movement skill program.* International Journal of Behavioral Nutrition and Physical Activity, 2010. **7**(1): p. 80.

18. Leslie, E., et al., *Community-based obesity prevention in Australia: background, methods and recruitment outcomes for the evaluation of the effectiveness of OPAL (Obesity Prevention and Lifestyle.* Adv Pediatr Res, 2015. **2**(23): p. 23.

19. [*https://www.hsph.harvard.edu/prc/projects/osnap/*](https://www.hsph.harvard.edu/prc/projects/osnap/). [cited 2019 01.12.19].

20. Swinburn, B.A., et al., *The Pacific Obesity Prevention in Communities project: project overview and methods.* Obes Rev, 2011. **12 Suppl 2**: p. 3-11.

21. [*https://healthy-ky.org/our-partnerships*](https://healthy-ky.org/our-partnerships). 18.07.24].

22. Moores, C.J., et al., *CONSORT to community: translation of an RCT to a large-scale community intervention and learnings from evaluation of the upscaled program.* BMC Public Health, 2017. **17**(1): p. 918.

23. Sutherland, R., et al., *A cluster randomised trial of an intervention to increase the implementation of physical activity practices in secondary schools: study protocol for scaling up the Physical Activity 4 Everyone (PA4E1) program.* BMC public health, 2019. **19**(1): p. 883.

24. Gonzalez-Viana, A., et al., *Promoting physical activity through primary health care: the case of Catalonia.* BMC Public Health, 2018. **18**(1): p. 968.

25. Rush, E., et al., *Project Energize: intervention development and 10 years of progress in preventing childhood obesity.* BMC Res Notes, 2016. **9**: p. 44.

26. QCWA Country Kitchens, T. *Queensland Country Womens Aassociation Country Kitchens*. 2017 [cited 2020 28/01/2020]; Available from: <http://www.qcwa.org.au/countrykitchens/meet-the-team/what-we-do/>.

27. Nothwehr, F., J. Andsager, and H. Haines, *The rural restaurant healthy options program: response of rural, local newspapers to a program press release.* Health Promot Pract, 2014. **15**(2): p. 217-22.

28. [*https://www.cdc.gov/span/php/about/past-span-programs.html*](https://www.cdc.gov/span/php/about/past-span-programs.html). 01.12.2020].

29. Stephanie Alexander Kitchen Garden Program, t. N.D. [cited 2020 28/01/2020]; Available from: <https://www.kitchengardenfoundation.org.au/content/about-us>.

30. Folta, S.C., et al., *National Dissemination of StrongWomen-Healthy Hearts: A Community-Based Program to Reduce Risk of Cardiovascular Disease Among Midlife and Older Women.* Am J Public Health, 2015. **105**(12): p. 2578-85.

31. [*https://www.superherofoodshq.org.au/*](https://www.superherofoodshq.org.au/). 01.12.2020].

32. Stevens, A.B., et al., *Increasing the Availability of Physical Activity Programs for Older Adults: Lessons Learned From Texercise Stakeholders.* J Aging Phys Act, 2016. **24**(1): p. 39-44.

33. [*https://slco.org/health/healthy-living/healthy-places/top-star/#:~:text=TOP%20Star%20stands%20for%20Teaching,assistance%20and%20resources%20to%20participants*](https://slco.org/health/healthy-living/healthy-places/top-star/#:~:text=TOP%20Star%20stands%20for%20Teaching,assistance%20and%20resources%20to%20participants)*.* 01.12.2020].

34. Beets, M.W., et al., *From policy to practice: strategies to meet physical activity standards in YMCA afterschool programs.* Am J Prev Med, 2014. **46**(3): p. 281-8.

35. YMCA Brisbane, t. *Schools' Breakfast Program*. 2018 [cited 2020 28/01/2020]; Available from: <https://www.ymcabrisbane.org/our-social-impact/schools-breakfast-program>.
