# Supplementary material for: Scale-up influences and definitions of scale-up ‘success’: evidence from globally scaled interventions
Source: Transl Behav Med. 2025 Feb 11;15(1):ibae063. doi: 10.1093/tbm/ibae063 (PMC11812037; doi:10.1093/tbm/ibae063)
Supplement: ibae063_suppl_Supplementary_File_3 [file ibae063_suppl_supplementary_file_3.docx]

**Participant Online Survey**

|  |
| --- |
| **Section 1: About you** |

The following section asks about your role and "**involvement**" with the initiative in question. By "**involvement**" we are referring to any formal or informal activities you undertook that were associated with the initiative. By "initiative" we are referring to the initiative or program named in your email.

1. **What is your name**

__________________________________________________________________________

1. **Please select your age range**

| 18-24 years |  |
| --- | --- |
| 25-29 years |  |
| 30-34 years |  |
| 35-39 years |  |
| 40-44 years |  |
| 45-49 years |  |
| 50-54 years |  |
| 55-59 years |  |
| 60+ years |  |

1. **Which of the following best describes your gender?**

| Male |  |
| --- | --- |
| Female |  |
| Other |  |
| I prefer not to say |  |

1. **Which organisation do you currently work for?** (If your organisation is based at multiple locations, please also specify which location you are based)

__________________________________________________________________________

1. **How long have you worked at this organisation?**

| Less than one year |  |
| --- | --- |
| 1-5 years |  |
| 6-10 years |  |
| 11-15 years |  |
| 16-20 years |  |
| 21-25 years |  |
| Over 25 years |  |

1. **Were you working for this organisation when you were involved in the initiative?**

🞏_1_ Yes

🞏_2_ No

**6a. If no, please state the name of the organisation(s) you were working for at the time:**

__________________________________________________________________________

1. **What is your current job role?**

__________________________________________________________________________

1. **Is this the same job role as when you were first involved in the** **initiative?**

🞏_1_ Yes

🞏_2_ No

**8a. If no, please state your job role(s) during the time you were involved in the initiative**

___________________________________________________________________________

| **Section 2: About the intervention** |
| --- |

*The following questions ask about the intervention and who was involved in scaling up. By “****initiative****” we are referring to the initiative or program named in your email.*

*By “****scaling up****” we are referring to deliberate efforts to increase the impact of successfully tested initiatives, to benefit a greater number of people and to foster policy and program development on a lasting basis.*

1. **What is the name of the initiative?**

__________________________________________________________________________

1. **Which of the following best describes the primary source(s) of funding for this initiative?** (Please select all that apply)

| a | Government |  |
| --- | --- | --- |
| b | Industry (e.g., Private for profit organisations, Health insurance companies, Sporting clubs) |  |
| c | Non-governmental organisations (NGOs) |  |
| d | Charity/philanthropic entities |  |
| e | International agency (e.g., World Health Organization, World Bank, UNICEF) |  |
| f | Other |  |

**If other, what best describes the primary source(s) of funding for this initiative?**

__________________________________________________________________________

1. **Approximately when did you first become involved with this initiative?**

____/____ (month/year)

1. **Which of the following best describes your PRIMARY role/involvement with the initiative?** (Please select all that apply)

| a | Design or development of the intervention components/strategies |  |
| --- | --- | --- |
| b | Testing the initiative in a research trial or pilot study |  |
| c | Evaluating impact or delivery of the initiative in practice (e.g. non-research trial) |  |
| d | Provision of funds to support research relating to the initiative |  |
| e | Advocacy or support for initiative implementation in practice |  |
| f | Provision of funds to support implementation or scale up in practice |  |
| g | Decision-making process to adopt or fund the initiative at a state/territory/national level |  |
| h | Overseeing or coordinating/supporting initiative delivery in practice |  |
| i | Other |  |

**If other, please state:**

__________________________________________________________________________

1. **Do you still have a role in this initiative?** (e.g., funding, delivery, evaluation)

🞏_1_ Yes

🞏_2_ No

**13a. If yes, please briefly describe ways in which you are still involved?** (e.g., types of activities you are involved in or responsibilities you have)

____________________________________________________________________________

**13b. If no, approximately when did your involvement with the initiative end?**

____/____ (month/year)

1. **Thinking about what the initiative involves and how it was designed and tested, how much do you agree with the following statements?**

|  | **The initiative …** | Strongly disagree | Disagree | Neither agree nor disagree | Agree | Strongly disagree | Don’t know |
| --- | --- | --- | --- | --- | --- | --- | --- |
| a | Was tested in a research trial and has evidence to show effectiveness |  |  |  |  |  |  |
| b | Was tested in a research trial and was shown to be feasible for delivery in the target setting |  |  |  |  |  |  |
| c | Has been advocated for by respected persons or institutions |  |  |  |  |  |  |
| d | Addresses a persistent or sharply felt problem in the community |  |  |  |  |  |  |
| e | Has a greater advantage in the target setting than existing practices |  |  |  |  |  |  |
| f | Implementation costs were counteracted by the potential health benefits gained |  |  |  |  |  |  |
| g | Could be easily integrated into existing practice and be understood, rather than being complex and complicated |  |  |  |  |  |  |
| h | Was compatible with users’ established values and norms |  |  |  |  |  |  |
| i | Aligned with state/territory/national strategies/policies at the time of scale up |  |  |  |  |  |  |

1. **Now think about the initiative scale up process more broadly. Based on your knowledge of the initiative in general (i.e. thinking beyond just the activities you were directly involved in), how much do you agree with the following statements?**

|  | **Prior to the initiative being delivered at scale…**  **(i.e. during scale up planning phases or when government support/funding was being sought)** | Strongly disagree | Disagree | Neither agree nor disagree | Agree | Strongly disagree | Don’t know |
| --- | --- | --- | --- | --- | --- | --- | --- |
| a | There was a focus on building capacity and ensuring sustainability within the target organizations/settings |  |  |  |  |  |  |
| b | Political support was established |  |  |  |  |  |  |
| c | Managerial infrastructures to support scale up were established |  |  |  |  |  |  |
| d | Human/budgetary resources and service components were established |  |  |  |  |  |  |
|  | **Since the initiative has been scaled up…**  **(i.e., delivered at a State, Territory or National level)** |  |  |  |  |  |  |
| e | Political support has been sustained |  |  |  |  |  |  |
| f | Managerial infrastructures to support scale up have been sustained |  |  |  |  |  |  |
| g | Human/budgetary resources and service components have been sustained |  |  |  |  |  |  |

| **Section 3: About the user organisation and resource team** |
| --- |

1. **The following question asks about user organizations.** *The “****user organization****” refers to the institution(s) or organization(s) that seek to adopt and implement the initiative.*

**How much do you agree with the following statements?**

|  | **Prior to scale up…** | Strongly disagree | Disagree | Neither agree nor disagree | Agree | Strongly disagree | Don’t know |
| --- | --- | --- | --- | --- | --- | --- | --- |
| a | Members of the user organization(s) perceived a need for the initiative |  |  |  |  |  |  |
| b | The user organization(s) had appropriate implementation capacity to deliver the initiative successfully |  |  |  |  |  |  |
| c | Timing and circumstances for scale up were appropriate for the user organization(s) |  |  |  |  |  |  |
| d | The user organization(s) possessed effective leadership and internal advocacy to support the initiative |  |  |  |  |  |  |

1. **Did the initiative involve a ‘resource team’ to facilitate scale up?**

*The “****resource team”*** *refers to the individuals and organisations that have either been involved in the development/testing of the initiative or seek to promote its wider use at scale (e.g. partnering stakeholders).*

🞏_1_ Yes

🞏_2_ No

🞏_3_ Don’t know

**17a. If yes, which individual(s) or organisation(s) were involved in scale up and what were some of the roles/activities undertaken?**

| **Name of individual or organization** | **Role or activity undertaken to support scale up** |
| --- | --- |
| 1. | 1. |
| 2. | 2. |
| 3. | 3. |
| 4. | 4. |
| 5. | 5. |
| 6 | 6 |
| 7. | 7. |
| 8. | 8. |
| 9. | 9. |
| 10. | 10. |

**17b. Drawing on your answers to the previous question, how much do you agree with the following statements?**

|  | **The resource team included individuals or organizations that….** | Strongly disagree | Disagree | Neither agree nor disagree | Agree | Strongly disagree | Don’t know |
| --- | --- | --- | --- | --- | --- | --- | --- |
| a | Were effective and motivated leaders who commanded authority |  |  |  |  |  |  |
| c | Understood the political, social and cultural environment in which scaling up took place |  |  |  |  |  |  |
| d | Had the ability to generate financial and technical resources |  |  |  |  |  |  |
| e | Had in-depth understanding of the user organization’s capacities and limitations, decision-making processes, key players and broader context |  |  |  |  |  |  |
| f | Had relevant technical/managerial skills and able to communicate strongly |  |  |  |  |  |  |
| g | Had relevant research and evaluation skills |  |  |  |  |  |  |
| h | Had the capacity to train members of the user organization to support/deliver the initiative appropriately |  |  |  |  |  |  |
| i | Had previous skills and experience with scaling up similar initiatives |  |  |  |  |  |  |
| j | Were involved in the research trial or pilot project testing |  |  |  |  |  |  |
| k | Were involved in designing the initiative |  |  |  |  |  |  |
| l | Were members of the user organization |  |  |  |  |  |  |
| m | Supported user organizations to ‘own’ and take responsibility for the intervention and scaling up process within their setting |  |  |  |  |  |  |

| **Section 4: Scale up strategy** |
| --- |

*The following section relates to the strategy used to scale up the initiative. The* *“****scale up strategy****”* *refers to* *plans and actions necessary to fully establish the initiative in policies and programs.*

*This includes channels that were used prior to scale up (i.e., during preparation and planning stages before the initiative is rolled-out at a state, territory or national level) and****during scale up****(i.e., when state, territory or national roll-out has taken place).*

1. **Which of the following channels were used to disseminate (spread) the initiative prior to and during scale up?**

|  |  | **Prior to scale up** | **During scale up** |
| --- | --- | --- | --- |
| a | Policy makers |  |  |
| b | NGOs or community-based organisations |  |  |
| c | The organisations or settings who would ultimately deliver the intervention |  |  |
| d | Community members/advocacy groups |  |  |
| e | Target recipients (i.e., end users) of the initiative |  |  |
| f | Other |  |  |
| g | None of the above |  |  |
| h | Don’t know |  |  |

**If other, please describe**

__________________________________________________________________________

1. **Which strategies were used to disseminate (spread) the initiative prior to and during scale up?**

|  |  | **Prior to scale up** | **During scale up** |
| --- | --- | --- | --- |
| a | Messages were tailored and used a format for each audience |  |  |
| b | Data was presented clearly, concisely and in a timely manner so that it was relevant and useable |  |  |
| c | Repeated messages were integrated into established communication networks of the user organisations |  |  |
| d | Barriers to effective communication were recognised and communication/marketing specialists were made use of |  |  |
| e | Other |  |  |
| f | None of the above |  |  |
| g | Don’t know |  |  |

**If other, please describe**

__________________________________________________________________________

1. **During scale up, were coalitions and networks generated to advocate for changes in policy or laws that were required for successful scale up?**

🞏_1_ Yes

🞏_2_ No

🞏_3_ Not applicable

🞏_3_ Don’t know

**20a. If yes, did this involve individuals or organisations outside of the government health sector?**

🞏_1_ Yes

🞏_2_ No

🞏_3_ Don’t know

1. **Were any of the following strategies used to enhance successful scale up within the user organisation(s)?** (Please select all that apply)

| a | Using online networks to communicate or support local implementation efforts | 🌕 |
| --- | --- | --- |
| b | Newsletters containing information regarding the intervention and/or implementation experiences | 🌕 |
| c | Providing resources to support implementation (e.g., information packs, toolkits, checklists) | 🌕 |
| d | Remote or online technical assistance/support services | 🌕 |
| e | Monitoring and providing feedback to organisations on implementation performance over time | 🌕 |
| F | Providing online training of target organisations and employees | 🌕 |
| G | Providing face-to-face training of target organisations and employees | 🌕 |
| h | Other | 🌕 |
| i | None of the above | 🌕 |
| j | Don’t know | 🌕 |

**If other, please describe**

__________________________________________________________________________

**22. Since the initiative has been implemented at scale (e.g. delivered and a State, Territory or National level), has it been evaluated?**

🞏_1_ Yes

🞏_2_ No

🞏_3_ Don’t know

**22a. If yes, approximately when have evaluations taken place?**

| **Month** | **Year** | **Evaluation conducted by (e.g., name of organisation)** |
| --- | --- | --- |
|  |  |  |
|  |  |  |
|  |  |  |
|  |  |  |
|  |  |  |
|  |  |  |

**22b. What were the outcome measures used in these evaluations? (e.g. process of implementation, health outcome, level of uptake)**

**22c. If yes, have the findings from this evaluation(s) been used to modify the scaling up process? (Please describe)**

_____________________________________________________________________________

**22d. If yes, have the findings influenced changes to policy or practice? (Please describe)**

_____________________________________________________________________________

**22e. If no, please describe why you think an evaluation has not been conducted?**

_____________________________________________________________________________

| **Section 5: Reflections** |
| --- |

1. **If a Government** supported, endorsed and/or funded the initiative for implementation, **what do you think were the**ir **main reasons**? (Please select all that apply)

| a | Aligned with a strategic priority of the Government | 🌕 |
| --- | --- | --- |
| b | Addressed a priority area of a specific Government Department | 🌕 |
| c | Aligned with existing institutional structures and historical policy trajectories. | 🌕 |
| d | Contributed towards meeting a relevant State/Territory/National target | 🌕 |
| e | Perceived to be technically and financially feasible within existing capacity and resource constraints | 🌕 |
| f | As a result of internal/external advocacy | 🌕 |
| g | An opportune policy window opened, or the political climate was right | 🌕 |
| h | Addressed a key need in the community | 🌕 |
| I | Other | 🌕 |
| j | Don’t know | 🌕 |

**If other, please describe**

__________________________________________________________________________

**24. Please describe any major barriers experienced during the scale up process**

__________________________________________________________________________

**25. Please describe any major facilitators experienced during the scale up process**

__________________________________________________________________________

**26. In general, how would you define that an initiative has been “successfully scaled-up”?**

__________________________________________________________________________

**27. Lastly, please list any other physical activity of nutrition related initiatives that you are aware of, which have been scaled up in your region/country at a state, territory or national level**

__________________________________________________________________________

**Thank you for completing this survey.**

**We greatly appreciate your time and effort to provide feedback.**
