# Supplementary material for: Scale-up influences and definitions of scale-up ‘success’: evidence from globally scaled interventions
Source: Transl Behav Med. 2025 Feb 11;15(1):ibae063. doi: 10.1093/tbm/ibae063 (PMC11812037; doi:10.1093/tbm/ibae063)
Supplement: ibae063_suppl_Supplementary_File_4 [file ibae063_suppl_supplementary_file_4.docx]

**Additional File 4.** Descriptive characteristics of survey participants

|  | **Academia (n=32)** | **Community (n=20)** | **Government (n=10)** |
| --- | --- | --- | --- |
| **Age (range)** | 30-34 years (1)  35-39 years (5)  40-44 years (3)  45-49 years (4)  50-54 years (4)  55-59 years (4)  60+ years (11) | 25-29 years (2)  30-34 years (5)  35-39 years (3)  40-44 years (1)  45-49 years (2)  50-54 years (3)  55-59 years (3)  60+ years (1) | 30-34 years (1)  35-39 years (2)  45-49 years (3)  50-54 years (2)  55-59 years (2) |
| **Sex** | Female (26)  Male (6) | Female (16)  Male (3)  Prefer not to say (1) | Female (4)  Male (5)  Prefer not to say (1) |
| **Time in organisation** | <1 year (1)  1-5 years (5)  6-10 years (5)  11-15 years (9)  16-20 years (6)  21-25 years (4)  >25 years (2) | <1 year (3)  1-5 years (7)  6-10 years (7)  11-15 years (1)  16-20 years (2) | <1 year (1)  1-5 years (3)  6-10 years (2)  11-15 years (2)  21-25 years (1)  >25 years (1) |
| **Time involved with intervention** | >1 <6 years (18)  >6 <10 years (3)  >10 <15 years (3) | <1 year (1)  >1 <6 years (8)  >6 <10 years (4)  >10 <15 years (3)  Prefer not to say (1) | >1 <6 years (2)  >6 <10 years (3)  >10 <15 years (1)  >15 years (2) |
