# Supplementary material for: Scale-up influences and definitions of scale-up ‘success’: evidence from globally scaled interventions
Source: Transl Behav Med. 2025 Feb 11;15(1):ibae063. doi: 10.1093/tbm/ibae063 (PMC11812037; doi:10.1093/tbm/ibae063)
Supplement: ibae063_suppl_Supplementary_File_5 [file ibae063_suppl_supplementary_file_5.docx]

**Additional File 5.** Percentage agreement of scale-up processes to the WHO ExpandNet framework core areas

| **WHO core area (Total components n=32)** | **Academia**  **Max n=32**  **N (%)** | **Community**  **Max n=19**  **N (%)** | **Government**  **Max n=11**  **N (%)** | ***Total N***  **Max n=62**  **N (%)** | ***P*** |
| --- | --- | --- | --- | --- | --- |
| **Intervention attributes (n=9)** | | | | |  |
| Was tested in a research trial and has evidence to show effectiveness | 25 (78) | 17 (90) | 9 (82) | 51 (82) | 0.611 |
| Was tested in a research trial and was shown to be feasible for delivery in the target setting | 28 (88) | 15 (79) | 9 (82) | 52 (84) | 0.651 |
| Was advocated for by respected persons or institutions | 30 (94) | 18 (95) | 9 (82) | 57 (92) | 0.459 |
| Addressed a persistent or sharply felt problem in the community | 31 (97) | 19 (100) | 9 (82) | 59 (95) | 0.104 |
| Had a greater advantage in the target setting than existing practices | 24 (75) | 17 (90) | 7 (64) | 48 (77) | 0.231 |
| Implementation costs were counteracted by the potential health benefits gained | 20 (63) | 15 (79) | 10 (91) | 45 (73) | 0.159 |
| Easily integrated into existing practice and understood, than complex and complicated | 25 (78) | 18 (95) | 9 (82) | 52 (84) | 0.372 |
| Was comparable with users' established values and norms | 25 (78) | 17 (90) | 9 (82) | 51 (82) | 0.611 |
| Aligned with state, territory or national strategies/policies at the time of scale up | 30 (94) | 16 (84) | 11 (100) | 57 (92) | 0.379 |
| **Intervention scale up process (n=7)** | | | | | |
| ***Prior to the intervention being scaled up:*** | | | | | |
| A focus on building capacity and ensuring sustainability within target organization/setting | 26 (81) | 16 (84) | 9 (82) | 51 (82) | 1.000 |
| Political support was established | 19 (59) | 11 (58) | 7 (64) | 37 (60) | 1.000 |
| Managerial infrastructures to support scale up were established | 16 (50) | 16 (84) | 11 (100) | 43 (69) | 0.001 |
| Human/budgetary resources and service components were established | 20 (63) | 16 (84) | 9 (82) | 45 (73) | 0.206 |
| ***After the intervention was scaled up:*** | | | | | |
| Political support was sustained | 13 (42) | 8 (42) | 4 (36) | 25 (41) | 1.000 |
| Managerial infrastructures to support scale up were sustained | 11 (36) | 14 (74) | 8 (73) | 33 (54) | 0.011 |
| Human/budgetary resources and service components were sustained | 10 (32) | 13 (68) | 6 (55) | 29 (48) | 0.038 |
| **User organisation attributes (n=4)** | | | | |  |
| Members of the user organisation(s) perceived a need for the initiative | 30 (94) | 17 (90) | 9 (82) | 56 (90) | 0.377 |
| Had appropriate implementation capacity to deliver the initiative successfully | 24 (75) | 16 (84) | 9 (82) | 49 (79) | 0.774 |
| Timing and circumstances for scale up were appropriate for the user organisation(s) | 25 (78) | 16 (84) | 9 (82) | 50 (81) | 0.911 |
| Possessed effective leadership and internal advocacy to support the initiative | 26 (81) | 15 (79) | 8 (73) | 49 (79) | 0.914 |
| **Resource team attributes (n=12)** | | | | | |
| Were effective and motivated leaders who command authority | 23 (89) | 13 (77) | 9 (82) | 45 (83) | 0.571 |
| Understood the political, social, and cultural environment in which scaling up takes place | 24 (92) | 15 (88) | 8 (73) | 47 (87) | 0.281 |
| Had the ability to generate financial and technical resources | 17 (65) | 13 (77) | 7 (64) | 37 (69) | 0.740 |
| Had in-depth understanding of the user organisation's capacities/ limitations, decision-making processes, key players and broader context | 23 (89) | 14 (82) | 11 (100) | 48 (89) | 0.380 |
| Had relevant technical/managerial skills and able to communicate strongly | 23 (89) | 15 (88) | 10 (91) | 48 (89) | 1.000 |
| Had relevant research and evaluation skills | 23 (89) | 14 (82) | 11 (100) | 48 (89) | 0.380 |
| Had capacity to train members of user organisation to support/deliver the initiative | 24 (92) | 15 (88) | 11 (100) | 50 (93) | 0.808 |
| Had previous skills and experience with scaling up similar initiatives | 17 (65) | 12 (71) | 8 (73) | 37 (69) | 1.000 |
| Were involved in the research trial or pilot project testing | 20 (77) | 12 (71) | 9 (82) | 41 (76) | 0.841 |
| Were involved in designing the initiative | 21 (81) | 13 (77) | 10 (91) | 44 (82) | 0.815 |
| Were members of the user organisation | 16 (62) | 11 (65) | 5 (46) | 32 (59) | 0.589 |
| Supported user organisations to take responsibility for the initiative/ scaling up process | 20 (77) | 15 (88) | 9 (82) | 44 (82) | 0.815 |

Fisher’s exact test used to assess differences in proportion of participant agreement between groups.
